# The effect of different estradiol levels on carotid artery distensibility during a long agonist IVF protocol

**DOI:** 10.1186/s12958-020-00608-w

**Published:** 2020-05-12

**Authors:** Jonna Leppänen, Kaisa Randell, Ursula Schwab, Jussi Pihlajamäki, Leea Keski-Nisula, Tomi Laitinen, Seppo Heinonen

**Affiliations:** 1 Department of Obstetrics and Gynecology, Kuopio University Hospital and University of Eastern Finland, Puijonlaaksontie 2, FIN-70210 Kuopio, Finland; 2 Obstetrics and Gynecology, University of Helsinki and Helsinki University Hospital, PO Box 140, HUS 00029 Helsinki, Finland; 3 School of Medicine, Institute of Public Health and Clinical Nutrition, University of Eastern Finland, Kuopio, Finland; 4 Department of Medicine, Endocrinology and Clinical Nutrition, Kuopio University Hospital, Kuopio, Finland; 5 Department of Clinical Physiology and Nuclear Medicine, Kuopio University Hospital and University of Eastern Finland, FIN-70210 Kuopio, Finland

**Keywords:** Carotid artery, In vitro fertilization, Distensibility, Estradiol, Infertility

## Abstract

**Background:**

This study was made to figure out, does low and high estradiol levels during in vitro fertilization (IVF) cycles have a different effect on carotid artery distensibility (Cdis), carotid artery diameter (Cdia), blood pressure and metabolic factors? Can the stimulation protocol be considered safe to women’s vasculature?

**Methods:**

We studied 28 women having a long agonist protocol IVF-treatment in Kuopio University Hospital during the years 2011–2016. Patients were examined at three time points: in the beginning of their own period (low estradiol), during the gonadotrophin releasing hormone (GnRH) analogue downregulation (low estradiol) and during the follicle stimulating hormone (FSH) stimulation (high estradiol). Women served as their own controls and their menstrual phase (2- to 5-day period after the beginning of menstruation with low estrogen) was used as the reference. Cdis and Cdia were assessed using ultrasound. Blood pressure, weight, estradiol levels and lipids were monitored.

**Results:**

Cdis, Cdia, systolic and diastolic blood pressures peaked during the GnRH-analogue treatment with the lowest estradiol levels. Cdis, Cdia and systolic blood pressures declined by 11% (*P* = 0.002), 3,8% (*P* < 0.001) and 2,5% (*P* = 0.026) during the FSH-stimulation when the estradiol levels were high. Cdis correlated significantly (*P* < 0.05) with systolic blood pressure, diastolic blood pressure and triglycerides in high estrogenic environment and with diastolic blood pressure (*P* < 0.05) when estrogen profiles were low.

**Conclusions:**

Carotid artery stiffens during the high estradiol levels compared to low levels and this was not explained by the higher diameter of the carotid artery, hyperlipidemia or blood pressure profiles. All the changes in Cdis and Cdia are variations of normal, and if there is no history of cardiovascular problems, it can be considered, that the stimulation protocol is not hazardous to vasculature.

## Background

Evidence on the effects of estrogen on arterial distensibility comes from three different sources: normal menstrual cycle, pregnancy and menopause.

In normal menstrual cycle estradiol levels measured at days 2–4 (menstrual phase), at follicular phase, at ovulatory phase, at early luteal phase and then, at late luteal phase have been 0.15, 0.18, 0.50, 0.47 and 0.24 nmol/l [1]. Three- to four-fold estrogen changes affected Cdis which changed significantly (up to 10%) depending on the menstrual cycle phase, increasing in the menstrual and follicular phase, peaking in the ovulatory phase and then, decreasing along the luteal phase [1].

In normal pregnancy estradiol levels increase about 100 times over non-pregnant levels, varying from approximately 4.6–7.3 nmol/l in the beginning of pregnancy (8 weeks) to 53.6–62.4 nmol/l in the end of pregnancy (40 weeks), [2]. Previously it has been shown, that along this 10-fold estrogen increase Cdis declined 36% from the first trimester to third trimester [3, 4], suggesting that an increase in a very high estrogen environment has opposite effects to those seen in normal menstrual cycle.

Estrogen levels decline in pre- and postmenopausal from approx. 0.51 to 0.07–0.11 nmol/l [5]. There is a trial made with postmenopausal women [6], some on placebo and others exposed to hormone replacement therapy (HRT). The estradiol level varied from 0.04 to 0.33 nmol/l (mean, placebo vs. HRT) and Cdis increased 32% in women using estradiol only and 40% women who received estradiol combined with progesterone.

In long agonist IVF protocol, very low estradiol level can be reached by using the GnRH analogue. Level of estradiol is increased to very high up, approximately in 1,5–2 weeks after starting the FSH. As a result, in long agonist IVF protocol women first have very low estradiol levels followed by very high levels.

The aim of the present study was to evaluate the changes in Cdis in a cohort of women undergoing a long agonist IVF protocol and having very different estradiol levels in their treatment cycles (first low followed by high estrogen). The hormonal intervention related to a long agonist IVF protocol gives us a good opportunity to study the effect of different hormonal statuses to vascular function from broader perspective. Some authors from our group have detected earlier same kind of findings about the hormonal changes to vascular function during pregnancy [3]. Is the stimulation protocol safe to vasculature, while the levels of estradiol vary from very low to high levels? Women served as their own controls and their menstrual phase (2- to 5-day period after the beginning of menstruation with low estrogen) was used as the reference. No earlier studies have been performed on Cdis variation in IVF-patients in whom the changes of normal menstrual cycle are in a way “exaggerated”. So, the results about the effect of hormonal changes to vascular function while long agonist protocol are novel. We also wanted to clarify the changes in Cdia, blood pressure and lipids in different hormonal phases.

## Methods

### Subjects

We prospectively studied 28 infertile women, ranging in age from 24 to 40 years (33 +/− 1 years, mean +/− SD), who had IVF treatment with a long agonist protocol in Kuopio University Hospital infertility clinic during the years 2011–2016. Women were recruited at the Kuopio University Hospital infertility clinic, when they visited doctor and IVF treatment was planned. The study population was planned to be 30 women, but two person’s blood samples were not taken in one visit, so they could not be included. The amount of women was estimated by the earlier studies made about Cdis during pregnancy [3, 4]. Before this study with the IVF patients, the expected change in vascular function could be anticipated by the changes which occurred during the pregnancy. From this basis the study population can be predicted.

### Study design

Women were encountered first time (visit 1) when they had own menstrual cycle without any hormonal treatment (from 2.- 5. days of menstruation). They started agonist medication, GnRH analogue (nafarelin acetate 800 mcg/day) and came to the next control, approximately 1 month later (visit 2) when their estradiol level was supposed to be very low, like after menopause. This time, they visited in order to start the controlled ovarian stimulation with FSH. The dose varied from 125 IU to 300 IU and was adapted to the patient’s body mass index (BMI), age and ovarian reserve (antral follicle count). The third visit (visit 3) was approximately 10 days later in the end of the IVF stimulation, when women had administered FSH at least for 9 days and the level of estradiol was supposed to be high. Blood samples were obtained and clinical measurements were done at every visit. For the hormonal intervention, primary outcome variables were Cdis and Cdia. Secondary outcomes were blood pressure and plasma lipid levels. If some data from those three visits was missing, the patient was excluded from the study.

### Carotid artery studies

Left carotid artery was scanned with an ultrasound according to standardized protocol using Sequoia 512 ultrasound mainframes (Acuson, CA, USA) and 14.0 MHz linear array transducer. 5-s cine loops were stored for subsequent offline analyses.

Cdis, Cdia and the intima-media thickness (IMT), were measured as previously described [7]. The common carotid diameter approximately 10 mm proximal to the carotid bifurcation w measured from B-mode images twice using the calipers of the ultrasound scanner. The means of the measurements were used as the end-diastolic and end-systolic diameters, respectively. In the results of this study, Cdia refers specifically to the end-diastolic diameter of the left common carotid artery. In the calculation of Cdis ultrasound imaging and concomitant brachial blood pressure measurements were used. Blood pressure was measured just before, and right after the carotid artery ultrasound scan, and mean of these results were used. Cdis was calculated as: ([systolic diameter – diastolic diameter]/diastolic diameter)/ (systolic blood pressure – diastolic blood pressure) [8]. To derive the maximal IMT, three measurements were performed. All carotid parameters were done by two different persons and the examiner did not know previous values, so all measurements were blinded.

Height and weight were measured and BMI calculated. The weight was measured by bioelectronic impedance analyzer (InBody 3.0; BioSpace, Seoul, Korea). Blood pressure was measured with an Omron M4i authomatic device (Matsusaka, Japan) for two times and the results were averaged.

Overnight fasting blood samples (12 h) were taken and analyzed in the laboratory. Samples were centrifuged at 2000 rpm for 10 min and serum plasma was separated. All lipid analyses were performed by standard methods by Konelab 60i Clinical Chemistry Analyzer (Thermo Electron Co., Waltham, MA, USA). The triglyceride concentration was determined by GPO-PAP enzymatic, photometric assay (Konelab Triglycerides kit, Thermo Electron Co.) and the total serum cholesterol concentration was analyzed by an enzymatic, photometric assay (Konelab Cholesterol kit, Thermo Electron Co.). Concentrations of high-density lipoprotein (HDL) cholesterol and low-density lipoprotein (LDL) cholesterol were determined by a direct, enzymatic, photometric method (Konelab HDL-Cholesterol and Konelab LDL-Cholesterol kits, Thermoelectron Co. Finland). Estradiol analyses were done by electrochemiluminescense immunoassay methods (ECLIA) with cobas e 601 analyzer (Hitachi High Technology Co, Tokyo, Japan). Reagent, which was used was Elecsys Estradiol II, (cat nro 03000079 190, Roche Diagnostics GmbH, Mannheim, Germany).

### Statistical analyses

Statistical analyses and calculations were done with SPSS for MAC, version 24 (SPSS Chicago, IL). First, Kolmogorov-Smirnov-test was done to test the normality of the distribution. Because in some of main variables (e.g. estradiol concentration) the distribution was skewed, the statistical analyses were done with the non-parametrical tests. Friedman’s test was used to discover the significance of differences between the tree visits. If the significance was found between three time points, then the Wilcoxon’s test was used to analyze differences between two visits. Spearman’s correlation analysis was used to test univariate associations. A *P*-value < 0.05 was considered statistically significant. Multiple regression analysis was not used in our study because observing several variables at the same analysis would need a bigger study sample. By using multiple regression analysis in our small study sample (28 women), it would be possible only use that kind of models, which could detect only one covariate at the time.

## Results

The clinical characteristics in visits 1, 2 and 3 are shown in Table 1.

**Table 1:** Clinical characteristics and responses to long agonist IVF protocol

	Visit 1	Visit 2	Visit 3	P-value
Age (Years)	33 ± 1	–	–	–
Gravity	0.4 ± 0.1	–	–	–
Para	0.2 ± 0.1	–	–	–
Height (cm)	166 ± 1	–	–	–
Weight (kg)	68.6 ± 2.6	–	–	–
Body mass index (kg/m2)	24.7 ± 0.9	–	–	–
Carotid distensibility (%/10 mmHg)	1.98 ± 0.49*	2.37 ± 0.55#	2.11 ± 0.50	0.002
Carotid diameter (mm)	5.1 ± 0.3§¤	5.2 ± 0.3†	5.0 ± 0.3	< 0.001
Systolic blood pressure (mmHg)	122 ± 11	122 ± 11‡	119 ± 11	0.026
Diastolic blood pressure (mmHg)	74 ± 7	76 ± 9	73 ± 9	NS
Total cholesterol (mmol/l)	4.7 ± 0.8║	4.9 ± 0.8†	4.5 ± 0.7	< 0.001
LDL cholesterol (mmol/l)	2.8 ± 0.7§■	3.0 ± 0.8†	2.5 ± 0.7	< 0.001
HDL cholesterol (mmol/l)	1.7 ± 0.3	1.8 ± 0.3	1.8 ± 0.3	NS
Triglycerides (mmol/l)	0.7 ± 0.2	0.7 ± 0.3	0.7 ± 0.3	NS
Estradiol (nmol/l)	0.2 ± 0.1*♦	0.1 ± 0.1†	6.2 ± 5.9	< 0.001
Estradiol (nmol/l)•	≤0.04–0.45	≤0.04–0.61	0.72–31.45	

The values are means±SEM

Significances: * *P* < 0.001 Visit 1 vs Visit 2, # *P* < 0.01 Visit 2 vs Visit 3, § *P* < 0.05 Visit 1 vs Visit 2, ¤ *P* < 0.05 Visit 1 vs Visit 3, † *P* < 0.001 Visit 2 vs Visit 3, ‡ *P* < 0.05 Visit 2 vs Visit 3, ║*P* < 0.01 Visit 1 vs Visit 2, ■ *P* < 0.01 Visit 1 vs Visit 3, ♦ *P* < 0.001 Visit 1 vs Visit 3, • A range of estradiol values

**Fig. 1:**
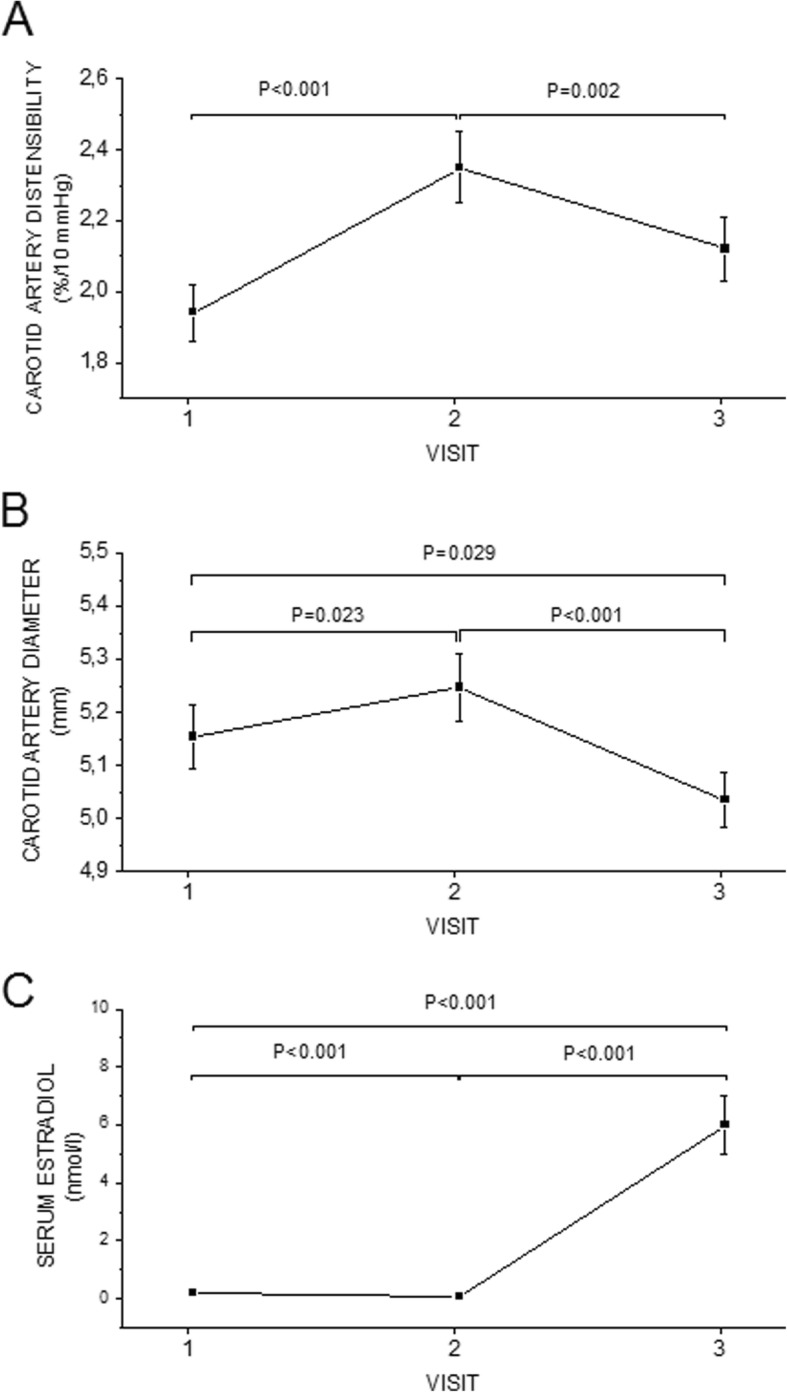
The values in Cdis, Cdia and serum estradiol levels in visits 1, 2 and 3

The values in Cdis, Cdia and serum estradiol levels in three different visits are shown in Fig. 1. Cdis value was at its highest level during the lowest, postmenopausal estradiol level in visit 2. Average Cdis became lower when the estradiol level was high in visit 3. Cdia was also largest during the visit 2 and the smallest in visit 3, when the estradiol level was high. The estradiol level was also low in visit 1, in the beginning of menstrual cycle and Cdia was higher also then than in visit 3. In this study, the lower the estradiol was, the better the Cdis and higher Cdia were. The IMT did not change significantly during the three visits (data not shown).

**Fig. 2:**
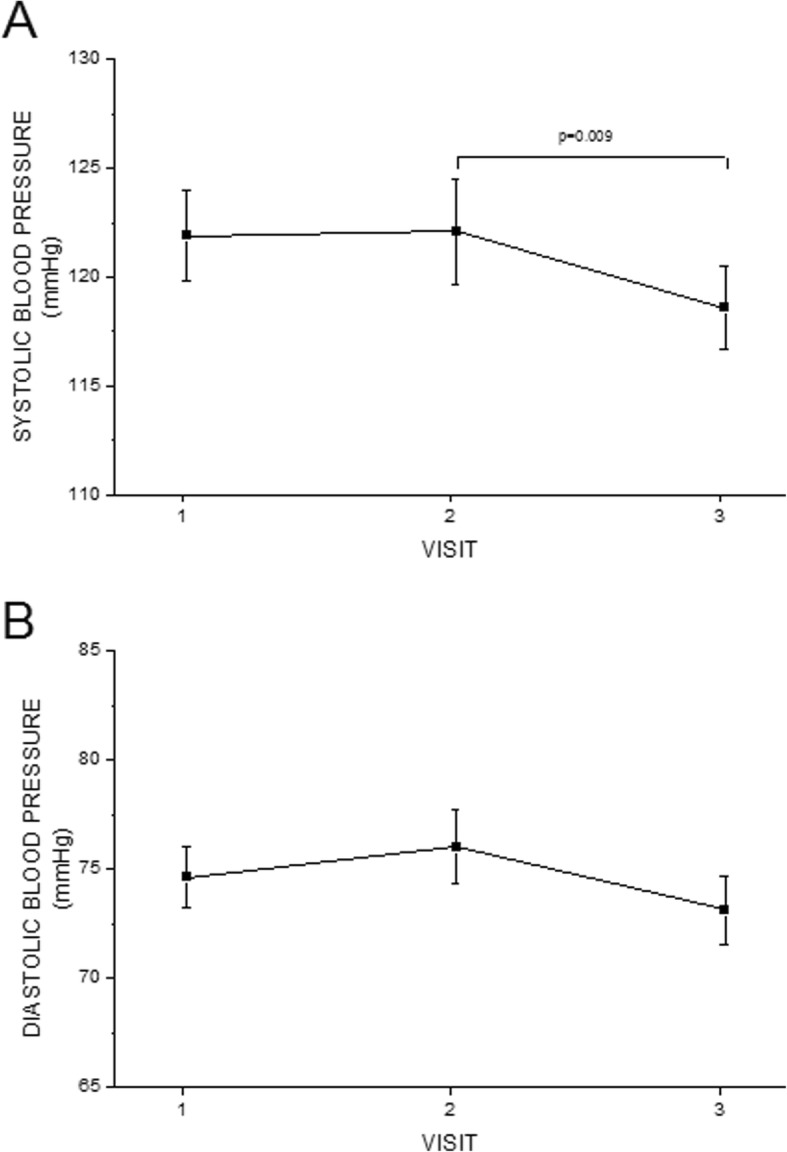
The values in systolic and diastolic blood pressures in visits 1, 2 and 3

Figure 2 shows the values of systolic and diastolic blood pressures in visits 1, 2 and 3. Both systolic and diastolic blood pressures were at highest level in visit 2, when the estradiol level was low. Vice versa, blood pressures were at lowest level in visit 3 during the high estradiol level.

Lipid profile changed through the visits 1, 2 and 3 (Table 1). Total cholesterol and LDL cholesterol were at their highest in visit 2 when the estradiol level was low. HDL cholesterol changed only a little and triglycerides remained the same.

Correlations between Cdis and selected variables are shown in Table 2. At the first visit Cdis correlated negatively with systolic blood pressure, diastolic blood pressure, weight, body mass index, total cholesterol, LDL cholesterol and triglycerides, while there were no statistically significant correlations between Cdis and Cdia, age, height, HDL cholesterol or estradiol level. At the second visit Cdis correlated with diastolic blood pressure, while there were no statistically significant correlations between Cdis and Cdia, age, systolic blood pressure, lipids or estradiol level. At the third visit, Cdis correlated with systolic blood pressure, diastolic blood pressure and triglycerides, while there were no statistically significant correlations between Cdis and Cdia, age, total cholesterol, LDL cholesterol, HDL cholesterol or estradiol level.

**Table 2:** Correlation between carotid distensibility and selected variables

	Carotid distensibility		
	Visit 1	Visit 2	Visit 3
Age	−0.17	–	–
Height (cm)	−0.22	–	–
Weight (kg)	− 0.42*	–	–
Body mass index (kg/m^2^)	−0.40*	–	–
Carotid diameter (mm)	−0.06	−0.15	− 0.20
Systolic blood pressure (mmHg)	−0.55**	− 0.32	−0.43*
Diastolic blood pressure (mmHg)	−0.51**	−0.40*	− 0.38*
Total cholesterol (mmol/l)	−0.41*	− 0.12	0.01
LDL cholesterol (mmol/l)	−0.42*	−0.29	− 0.09
HDL cholesterol (mmol/l)	0.06	0.33	0.24
Triglycerides (mmol/l)	−0.45*	−0.33	− 0.43*
Estradiol (nmol/l)	0.03	−0.25	0.19

The values are Spearman’s correlation coefficient r

Significances: * *P* < 0.05, ** *P* < 0.01

The only statistically significant correlation for Cdia was found at the third visit between Cdia and age (*r* = − 0.407, *P* = 0.032).

## Discussion

The main finding of this study was that Cdis declined 11% on an average while estradiol levels increased from 0.1 nmol/l to 6.2 nmol/l (mean) concentration (62-fold) in their IVF cycles. Correlation analyses showed that this decline was not due to the higher diameter of the carotid artery, high blood pressure or dyslipidemia. We conclude, that the estradiol level is likely to contribute to the function of carotid artery and blood pressure and despite of these hormonal changes, the stimulation protocol is not hazardous to women’s vasculature. All the changes in Cdis are variations of normal.

Estrogen is likely to play a role in arterial distensibility, but the available evidence suggests that its effect depends on the baseline estrogen level and magnitude of the estrogen concentration changes. In normal menstrual cycle the estrogen change is proportionately moderate (three to four-fold) from low baseline levels. This results in 10% increase in Cdis [1]. In pregnancy from the first to third trimester the estrogen change is from high baseline to even ten-fold higher concentration in the third trimester, which in turn decreased carotid artery elasticity by one third (36%) [3, 4]. In menopause, the change is from low to very low estrogen and HRT rises it close to premenopausal level, with an improvement of 30 to 40% in Cdis with replaced estrogen [6].

In the current study setting estrogen changes are exaggerated compared to natural menstrual cycle, since levels at the ovulatory peak are up to 60-fold higher than during suppression and up to 10-fold higher than in natural cycle. Interestingly, in this setting carotid artery elasticity declined, implying that an IVF cycle is very different from natural cycle in terms of carotid artery elasticity. This phenomenon, however, is very similar that seen in course of normal pregnancy. It may be speculated that beyond certain estrogen levels, its effect is opposite to those seen at low or moderate levels. It has been previously speculated, that this phenomenon can be adaptation to the altered hemodynamics occurring during pregnancy [3] and also, that there are less estrogen receptors occurring in the carotid arteries and this could explain the difference in Cdis compared to other arteries [9].

It has been published earlier [10], that there is a complex relation of estrogen with arterial health, higher estradiol was associated with smaller Cdia, but lower Cdis. That study was made with postmenopausal women, but the findings are in concordance with ours. However, among published studies the results have been controversial [11].. There are also studies made with postmenopausal women with HRT and its effect to the Cdis. One of them [6] discovered, that carotid arterial compliance and Cdis were significantly higher and stiffness index values lower with HRT compared to placebo treatment. Two other studies had similar findings [12, 13]. A study made with perimenopausal women [14] and a study made with postmenopausal women [15] found out, that there was no effect on Cdis with HRT versus placebo using.

The clinical implications of the present results can be seen via stimulation protocol’s impact to vascular health. The huge variation in estradiol levels during a long agonist IVF treatment gives an opportunity to study this phenomenon and effect to vasculature in vivo. During the stimulation, the exposure for high estradiol is after all quite short. In general, low Cdis is associated with greater stroke risk [16]. In our study, Cdis lowered during the highest estradiol level. Still, the Cdis values stayed within the normal limits [17] and Cdis lowered only − 0.26 SD (Fig. 1). The change of Cdis can be detected between visits 2 and 3, but this finding seems to be only modest and is likely to be clinically less significant. We speculate that if there are plenty of other risk factors for stroke, then this phenomenon could additionally have unfavorable effect. Furthermore, although in short term the values of Cdis do not differ much from baseline values, hormonal status may contribute to Cdis and this might have consequences to vascular health in long term. This study shows, that the stimulation protocol is not hazardous to vasculature, if there are no earlier cardiovascular problems. Further studies are needed to clarify more this phenomenon. It has been described earlier, that there will be regional heterogeneity in elastic behavior of the arterial tree with aging and hypertension, there will be vascular remodeling [18]. It has also been shown, that carotid artery stiffens during pregnancy [4]. This finding is in line with ours.

The widening of the arteries is related to a worse cardiovascular profile, active coronary plague volume and cardiovascular events [19]. In our study, Cdia was highest during the low estrogen level and then diminished during the high estrogen level, and this could be considered as a beneficial finding related to estrogen when considering the cardiovascular health. It has been reported [20], that arterial lumen widens with age in normotensive postmenopausal women and that might compensate age-related increase in arterial stiffness. The mechanism may be different in young women [21]., that menopause may increase aortic stiffness, which probably causes the rise in systolic blood pressure and this may lead to a slight dilatation of common carotid artery. They suggested that aortic elasticity diminishes due to menopause and aging.

Many studies have shown [6, 22–24], that estrogen deprivation may increase blood pressure. Also in our study, blood pressure was at its highest during the low estradiol level and lowered during the high estradiol level, which could be considered as an advantageous finding, even when the change in blood pressure was only 3 mmHg between visits 2 and 3 (Table 1). That probably does not have that much clinical significance, but a trend can be seen. Conversely it has been shown, that brachial arterial blood pressure did not change significantly during the menstrual cycle in young women [1].

In our study, lipid profile was at its worst during the low estradiol level in visit 2 and then got better during the high estradiol level, so total cholesterol and LDL were higher during the low estradiol level and then improved during the IVF stimulation. During such a short period this phenomenon does barely have any clinical significance, but there was an association between Cdis and lipids. Diet barely has an impact to those findings while this short period of observation.

The validity of this study can be considered good since all women underwent the same IVF protocol in one unit and they served as their own controls. Laboratory tests were analyzed blindly and the Cdis and Cdia were measured without the information of IVF cycle phase. The cause of underlying infertility unlikely plays a role in arterial elasticity as all women went through the same treatment protocol. One limitation of this study was a small study population. Limited statistical power in analyses can affect our results, and when interpreting the results of this study, a possibility for type two error should be taken into consideration. Considering the methods, Cdis-assessment is likely to be sensitive, because we could detect several previously known and clinically relevant associations between Cdis and systolic blood pressure, diastolic blood pressure, dyslipidemia and obesity.

## Conclusions

Our findings reveal, that carotid artery stiffens during the high estradiol levels compared to very low levels and this is not due to the higher diameter of the carotid artery, high blood pressure or hyperlipidemia. This phenomenon is important considering to vascular health. All the changes in Cdis are variations of normal, so the stimulation protocol can be considered safe to vasculature despite variations in estradiol levels, if there is no history with cardiovascular problems. However, further studies are needed to clarify more about this phenomenon.

## Data Availability

The datasets generated and analyzed during the current study are not publicly available due they include patient’s personal data, but are available from the corresponding author on reasonable request.

## Abbreviations

BMI: Body mass index
Cdia: Carotid artery diameter
Cdis: Carotid artery distensibility
FSH: Follicle stimulating hormone
GnRH: Gonadotrophin releasing hormone
HDL: High - density lipoprotein
HRT: Hormone replacement therapy
IMT: Intima - media thickness
IVF: In vitro fertilization
LDL: Low - density lipoprotein
